# A synthetic synthesis to explore animal evolution and development

**DOI:** 10.1098/rstb.2020.0517

**Published:** 2022-07-18

**Authors:** Mindy Liu Perkins, Lautaro Gandara, Justin Crocker

**Affiliations:** Developmental Biology Unit, European Molecular Biology Laboratory, 69117 Heidelberg, Germany

**Keywords:** synthetic biology, biophysical constraints, evolvability, gene regulatory network, enhancer, phenomics

## Abstract

Identifying the general principles by which genotypes are converted into phenotypes remains a challenge in the post-genomic era. We still lack a predictive understanding of how genes shape interactions among cells and tissues in response to signalling and environmental cues, and hence how regulatory networks generate the phenotypic variation required for adaptive evolution. Here, we discuss how techniques borrowed from synthetic biology may facilitate a systematic exploration of evolvability across biological scales. Synthetic approaches permit controlled manipulation of both endogenous and fully engineered systems, providing a flexible platform for investigating causal mechanisms *in vivo*. Combining synthetic approaches with multi-level phenotyping (phenomics) will supply a detailed, quantitative characterization of how internal and external stimuli shape the morphology and behaviour of living organisms. We advocate integrating high-throughput experimental data with mathematical and computational techniques from a variety of disciplines in order to pursue a comprehensive theory of evolution.

This article is part of the theme issue ‘Genetic basis of adaptation and speciation: from loci to causative mutations’.

Life is infinitely stranger than anything which the mind of man could invent.

– Sherlock Holmes, ‘A Case of Identity’ in *The Adventures of Sherlock Holmes* (1891)

## 1. Introduction

Over a century of advances in molecular techniques have gifted evolutionary biologists greater access to the source of heritable variation than Mendel or Darwin could have imagined (table 1). We are only now beginning to understand what processes and principles convert an enormous diversity of genotypes into an even greater diversity of phenotypic forms. In the search for general principles of animal evolution, researchers have found that evolution is driven both by mutations to proteins and mutations to regulatory regions. Although it has been proposed that mutating *cis*-regulatory regions may avoid the pleiotropic effects of mutating proteins [7], recent studies indicate that modifying transcriptional enhancers may also introduce extensive pleiotropy [8]. Indeed, evolutionary innovations in regulatory regions have been found to affect every level from morphology to physiology to behaviour [6] even when target proteins are functionally conserved [9–14]. These observations highlight that a comprehensive evolutionary theory necessitates functional understanding of how regulatory regions orchestrate the activities of cells, tissues, and ultimately whole organisms.

**Table 1.** Defined terms.

synthetic biology	Creating biological systems in order to establish control over cellular behaviours (modified from Bashor & Collins [1]).
synthetic approach	Introducing an artificial or constructed element into a biological context; e.g. introducing mutations/duplications/indels, genetically engineered constructs (including circuits) (see also Garcia *et al.* [2]).
genotype-to-phenotype map	Sum of ways by which genotypic information influences the phenotype of an organism (adapted from Houle [3]).
phenomics	Acquisition of high-dimensional phenotypic data on an organism-wide scale. While genomic methods can aspire to survey genetic information comprehensively, the vast information content of phenotypes prevents their exhaustive characterization. Phenomics, instead, relies on prioritizing what to measure (adapted from Houle [3]).
enhancer	A contiguous DNA segment capable of boosting transcription from the promoter of a target gene, which could be located thousands of base pairs away. Enhancers can be found upstream or downstream to their target promoter and can even be located within transcriptional units.
regulatory region	DNA sequence that alters the expression of target genes. In this review, we will use the term primarily to refer to enhancers, promoters, silencers and insulators, which function through the binding of transcription factors or other regulatory molecules to DNA.
gene regulatory network	Set of transcription factors and signalling molecules that interact with each other and with DNA to regulate the expression of a set of genes (some of which may encode the transcription factors themselves).
complexity	Has many precise mathematical definitions in different contexts and fields, related in some way to the ease or difficulty of describing a given structure. For our purposes, the ‘complexity’ of a system scales with the number of potential behaviours that the system could demonstrate; e.g. for networks, ‘complexity’ roughly scales with size (number of elements and interactions between them).
modularity	The degree to which a network can be divided into independent subnetworks responsible for executing particular functions.
robustness	The degree to which a system is sensitive to perturbation or variation in architecture, environment, noise, parameters, etc. [4]. It can be precisely defined depending upon the performance measure of interest [5], though its usage in biological literature is not standardized, partly because it is rarely quantified.
evolvability	The capacity of a population to produce the heritable phenotypic variation of a kind that is not unconditionally deleterious (adapted from Masel & Trotter [6]).

We face a number of fundamental challenges to elucidating phenotypic evolution through regulatory mutations. On a very basic level, it is still difficult to predict gene expression levels from an arbitrary promoter by an enhancer of known sequence. Recent progress in mathematical and mechanistic modelling has introduced general thermodynamic frameworks for the interactions of regulatory factors with enhancers [15], and reliable quantitative predictions have been produced for specific cases in developing organisms (e.g. [16,17]). However, we have yet to generate models approaching the accuracy and generalizability of comparable theories for prokaryotic gene expression, which is itself still an active area of research beyond certain controlled contexts [18].

Additionally, we have only a limited empirical understanding of the possible paths for regulatory evolution. Most evidence derives from either observational measures of standing variation or biased perturbations of genomic regions that are already known to bind transcription factors strongly. This methodological skew reflects a general trend of relying more on genome-scale experimental data than would be typical for focused experimental studies [19]. Even when key regulatory regions are positively identified, their genetic variants may contribute very little to measurable phenotypic differences [20,21] or trigger cascading effects that result in pleiotropy [22–24]. Both of these phenomena emerge in part through complex interactions across organizational scales from cells to tissues to organs, each of which is subject to selection pressures [25]. Thus, cultivating a causal understanding of evolution will entail transferring insights from one level of biological organization to another with predictive power and, ideally, interpretability.

**Figure 1.**
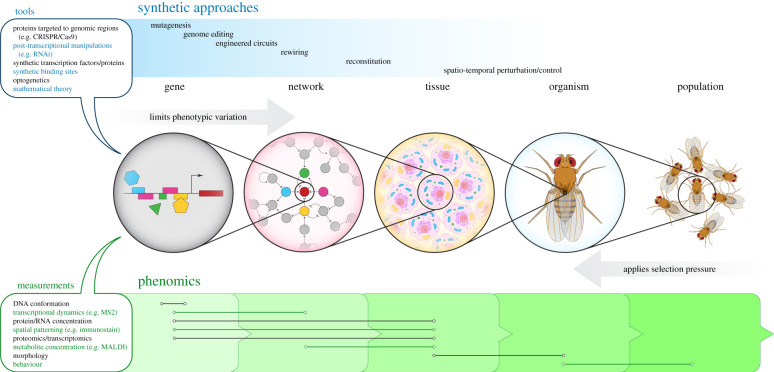
Synthetic approaches coupled with multi-level phenotypic measurements (phenomics) can characterize interactions across biological scales, which shape the possible and actual behaviours of living systems. Lower levels of organization tend to limit phenotypic variation at higher levels, while functional needs at higher levels may impose selection pressures on lower levels. Both processes shape evolvability.

Here, we discuss gene regulation as a motivating example of how synthetic approaches can uncover evolutionary principles across biological scales. Many of these approaches are borrowed from synthetic biology, an interdisciplinary field that emphasizes rationally designing organisms to execute predefined functions, predicated on a practical understanding of the genetic and biophysical causes behind phenotypic features. Recent advances in synthetic technologies and theories have targeted throughput and control across multiple levels of biophysical and functional organization [1,26]. We outline how these developments are furnishing experimental evolution with the very toolkit it needs to chart a quantitative course from genotype to phenotype (figure 1).

## 2. Synthetic approaches to manipulating regulatory regions

Studies of transcriptional regulation often focus on developmental enhancers, which specify the precise time, level and location of gene expression during embryonic growth [27] and therefore act as major contributors to phenotypic evolution [27,28]. We now have the ability to map millions of putative enhancer regions across genomes [29], but defining which of these sites are functional remains a challenge. Inversely, we cannot yet reconstitute even the most well-characterized developmental enhancers from synthetic binding sites [30,31]. Deriving a causal relationship between regulatory sequences and phenotypes will require a better understanding of the molecular mechanisms driving precise patterns of gene expression, as well as the consequent roles of cryptic variation and ‘robustness’ [32,33] on enhancer evolution. We propose that synthetic approaches, which involve introducing artificial elements into a biological context, enable experimental exploration of possibilities unsampled by natural evolutionary processes, while simultaneously granting the high levels of precision and control that are necessary to resolve causal factors across scales.

We start by exploring how synthetic approaches may clarify regulatory logic. Mutational scanning provides a powerful means to assay the activities of regulatory elements and to explore evolutionary potential [34–38]. These studies involve screening an unbiased assay of genetic variants and can therefore identify evolutionarily relevant sequences that are not surfaced by genomic or designed-sequence approaches based on previously identified binding sites [39]. In particular, mutational scanning experiments have uncovered non-canonical and low-affinity binding sites [39–41] that appear in appreciable frequencies in natural populations [42], as well as rare or deleterious mutations that are difficult to observe *in vivo* [43]. A recent study in *Drosophila melanogaster* used an automated robotics pipeline to survey an unbiased mutation library for a developmental enhancer, finding that almost all mutations altered gene expression and that the location and levels were highly interdependent [44]. The results are consistent with mammalian studies including both developmental enhancers [45] and promoters [46], indicating that widespread pleiotropic effects may constrain the evolvability of developmental enhancers [47,48]. In the future, mutational scanning could be used to define the essential features of enhancers and inform predictive models of enhancer function.

Most studies such as the one just described rely on reporter constructs to evidence phenotypic responses to genotypic changes. While reporter gene-based assays can provide information on the activity of individual regulatory regions, there are several potential caveats to generalizing the conclusions. First, removing an enhancer from its native location and placing it in a novel chromatin environment may facilitate the binding of transcription factors to sites that are not used in their native context [49]. Second, many reporter constructs are based on heterologous promoters, which may augment gene expression relative to the endogenous promoter [50] or behave differently from the native locus if the distance (in bp) from promoter to enhancer is not retained. Third, what is identified as the enhancer (and therefore included in the reporter construct) may miss essential components regulating the target promoter, including binding sites adjacent to the native enhancer [51–53] or additional enhancers located elsewhere in the genome [54–58], which can buffer the phenotypic effects observed from mutations to the construct. Finally, in some cases, it is not even possible to isolate individual regulatory regions that drive reporter gene expression [59,60], emphasizing the need to explore beyond minimal, ‘modular’ enhancers.

Classical reporter-gene assays can be complemented by a number of methods and techniques from synthetic biology for interrogating native loci. Unlike screens performed in transgenes, assays that modify endogenous sequences can be used to directly test the physiological effects of different genetic variants. This capability is crucial given that changes in gene expression levels alone are not necessarily correlated with changes in evolutionary fitness [61]. New technological advances will facilitate building mutagenesis libraries in endogenous loci similar to the ones that rely on reporter-gene assays. Genome-editing tools, including the CRISPR-associated protein 9 (CRISPR-Cas9) system, have been used for high-throughput loss-of-function screens of regulatory elements [62–68]. CRISPR-Cas nucleases introduce insertion and deletion (indel) mutations of variable length, increasing the scope of genetic variations that can be tested relative to conventional mutagenesis techniques. CRISPR-Cas9 and other DNA-binding proteins such as TALE-effectors can also be fused to activator and repressor domains and then targeted to specific enhancers in order to modulate gene expression *in vivo* [69–73]. Activator and repressor domains may remodel chromatin structure across hundreds of nucleotides [71], introducing larger perturbations than CRISPR-Cas nucleases and thereby improving enhancer detection.

Coupling synthetically perturbed enhancers with live imaging holds enormous potential to shed light on how enhancers establish precise expression patterns and confer phenotypic robustness. Recent technological advances have enabled real-time imaging of transcription by tagging mRNA with multiple repeats of a stem-loop sequence that is recognized by a binding protein fused with a fluorescent protein. The resulting ‘transcription spots’ are then visualized using standard live microscopy techniques [74], revealing the precise timing and spatial location of gene expression. For example, the *even-skipped stripe two* (eveS2) enhancer, one of the most well-characterized developmental enhancers [75], is active across a short window of time in the early embryo [76] and drives highly dynamic spatial patterns of expression [77]. Simultaneous imaging of transcription factors and regulatory targets [78] will be particularly instructive for probing the regulatory logic of individual loci as well as interactions among transcription factors.

We have presented here just a few of the available techniques for modifying regulatory regions and measuring their effects. Future efforts in technological development should focus on comprehensive approaches to interrogating regulatory elements in their native context. Analysis of the resulting data will be facilitated by our enhanced capacity to detect genetic variants with small effect sizes, as well as continued improvements to the sensitivity and throughput of live-imaging methods. With a combination of these approaches, we may begin to tease out the precise relationships between regulatory sequences, transcription factors and the expression patterns of target genes—the first step toward understanding the potential of genetic variants, realized or unrealized, to influence downstream phenotypes.

## 3. Form and function in gene regulatory networks

Synthetic approaches suitable for individual regulatory regions naturally extend to the study of gene regulatory networks (GRNs), or functional groupings of genes that produce phenotypes from genotypes by modulating expression levels in response to internal and external signals. Even small GRNs can execute involved processes such as disturbance rejection [79] and fold-change detection [80] over timescales much longer than the few seconds required for transcription initiation [16]. Although complex functions require complex networks, simple functions do not require simple networks. Rather, the same network structure may perform multiple functions depending on context or parameters, and the same function may be performed by multiple networks. These observations underlie nearly every aspect of network evolution, from the emergence of complexity through non-adaptive mechanisms [81,82] to the prevalence of ‘network drift’ within and between related species [83–86]. Classic challenges to the evolutionary study of GRNs involve their identification, level of conservation and ease of emergence or ‘rewiring’ based on mechanisms of binding site or enhancer turnover [12,13]. The synthetic biological emphasis on mathematical ‘design principles’ provides a fresh perspective from which to consider these challenges.

At the level of networks, understanding causal relationships between genotypes and phenotypes is complicated in a number of ways. If a regulated gene is itself a transcription factor, then downstream genes may constrain the minimum or maximum expression of the gene [87]. Compounded constraints for genes involved in multiple pathways may result in the emergence of non-adaptive features [88]. Even genes that are not directly interacting can become coupled through biophysical processes. For example, crosstalk, in which regulatory factors bind nonspecific or multiple targets, introduces a tradeoff between site specificity and the number of regulatory targets [89] as well as pressure to reduce active regulation [90], among other theoretical implications [91–93]. Given the high potential for coupling among GRNs, it is perhaps little surprise that researchers have proposed an ‘omnigenic model’ in which essentially all genes with regulatory variants contribute to complex traits [24].

Synthetic biologists have developed a toolkit sufficient for constructing entirely synthetic GRNs from engineered enhancers, promoters and transcription factors in bacteria and eukaryotes [94]. Considerable conceptual and experimental effort has aimed at understanding the reliability and robustness of these circuits, with a recent focus on predicting [95–99] and mitigating [100,101] the impact of crosstalk and resource sharing [1,102,103]. The tools and theories developed to design GRNs are grounded in approaches from engineering and physics, and hold enormous potential for helping evolutionary biologists to make sense of network-level changes over time.

For example, networks are subject to hard limits on their functionality that shape, directly or indirectly, the optima of the fitness landscape. Mathematical theory has elucidated fundamental limits to such functions as noise suppression [104], fold-change detection [105] and chemical sensing [106]; uncovered insurmountable tradeoffs among aspects of GRN performance [107–109] that may depend on system size [110]; and identified schemes that optimally achieve desired behaviours [111,112] under relevant constraints [113]. How closely networks operate to their theoretical optima—and how closely they can be brought to approach these optima through experimental evolution or synthetic recapitulation—may inform our understanding of selection pressures, adaptive responses and the balance between necessity and sufficiency.

Similarly, both evolutionary and synthetic biologists have investigated the degree to which GRNs are modular [114]—the former to determine whether decentralized control confers adaptive advantage [82,115–119], the latter to simplify the analysis and design of sophisticated circuitry [1,120], and both with the hope of improving human interpretability of complex structures. In principle, modules can be any size, which has historically biased research toward small, prevalent architectures or ‘motifs’ that are relatively simple to model, build and interpret [121–123]. Regardless of how a particular module is identified, its evolvability is strongly constrained by the pattern of regulatory interactions (topology) [124], especially when an original function must be preserved even as novel phenotypes arise [125–128]. Experiments with synthetic rewiring in bacteria have shown that topological differences can cause modules with the same average behaviour to diverge in their responses to stochastic fluctuations in chemical concentrations [129]. Moreover, the *strength* of regulatory interactions typically determines module behaviour, such that modules may vary in their robustness to enhancer mutations or noisy gene expression depending upon their quantitative biophysical properties [5,128,130–132].

The degree to which modules are (i) separable from the context of the full GRN or whole cell and (ii) sensitive to variation in topology or biophysical parameters begs a host of evolutionary questions. Do less parameter-sensitive modules exhibit greater standing variation? Are parameter-sensitive modules more likely to be evolutionarily co-opted? How do the relative sensitivities of overlapping modules determine evolvability [133–136]? To what extent does the parameter sensitivity of a module constrain the remainder of the network to maintain its own narrow operating range? Addressing these questions would help clarify when fitness benefits derive from ‘internal’ improvements in the function of a module or ‘external’ improvements in the behaviour with respect to other modules [137].

A straightforward way to proceed involves using the techniques discussed in the previous section to simultaneously disturb multiple regulatory regions belonging to the same GRN. It may be difficult, however, to infer causation from experiments conducted on complex and incompletely characterized endogenous systems. A complementary approach is to explore evolutionary principles in simple synthetic networks whose constituent parts can be individually measured and manipulated, and whose functions are understood *a priori*. For example, it has been shown that the set of regulatory interactions among three genes in a synthetic circuit biases the phenotypic variation observed after random mutation to the regulatory regions, even if the initial phenotypes are identical [138]. Similar techniques could be used to quantify dynamical behaviours and probe interconnections of modules, yielding invaluable predictive insights into the network-level effects of enhancer variation. The degree to which synthetic networks can be made orthogonal to endogenous pathways will present challenges to ‘parallel evolution’ experiments, but could also be deliberately employed to investigate the evolutionary implications of resource sharing, dosage change or crosstalk, which have been proposed to act as selective pressures against complexity [92].

Truly leveraging insights from simple synthetic modules will require theoretical and experimental work to predict the behaviour of full networks from constituent components under interconnection [139]. Such a programme necessitates ‘parts characterization’ of single loci or entire modules, in line with historical engineering practices and repeated calls for standardization across synthetic biology [1,26]. Most existing research has been carried out in microbes (e.g. [18]), but future efforts should also focus on multi-cellular sexually reproducing eukaryotes in particular, as to the best of our knowledge very little research has dealt with the implications of enhancer heterozygosity for network function [140], the interaction of population size with network evolution [115,141], or the role of ‘multiple inheritances' in generating variation in network architecture and thus phenotype [137,142]. Whether disrupting endogenous networks or constructing synthetic ones, experiments linking *cis*-regulatory variation to the output of GRNs will require new techniques and tools capable of manipulating and monitoring multiple genes simultaneously. Such detailed study of regulatory regions in context will be essential for understanding biophysical and evolutionary constraints, and therefore for predicting phenotypic consequences on larger organizational scales.

## 4. Leveraging phenomics to explore animal evolution and development

Historically, most efforts to study adaptation have involved providing lists of genetic variants that influence the phenotype of interest. Large-scale approaches such as genome-wide association studies identify regions correlated with the observed phenotypic variation, but often fall short of functional validation [21,143]. This issue, commonly referred to as *the missing heritability problem* [143], is often assumed to reflect the wide distribution of genes and regulatory regions along the genome [24] that limits the explanatory potential of genomic approaches. The problem could also arise, however, from the failure of genomic approaches to consider contextual factors, such as environmental stimuli or feedback loops between phenotypic levels, that also influence organismal behaviour. Therefore, a complete genotype-to-phenotype map will require not only a comprehensive account of genetic diversity, but also (i) a high-dimensional, multi-scale depiction of the phenotypic space (phenomics), (ii) a standardized set of techniques borrowed from synthetic biology for targeted manipulation of the system of study and (iii) a systematic exploration of environmental conditions, closer to native ecological settings where food shortages or extreme temperatures are plausible scenarios.

Phenomic characterization is becoming more feasible as high-throughput phenotyping technologies become more available, ranging from transcriptomics and epigenomics to proteomics and metabolomics. Techniques that can link single-cell data to tissue architecture will be particularly useful, as bulk datasets tend to hide effects that are restricted to specific cell types or regions but that could deeply affect organism-level, fitness-related phenotypes such as mating behaviours or survivability. Recently, single-cell RNA-seq has been performed on relatively large samples from various species [144–146] including humans [147,148], revealing significant levels of cell-to-cell gene expression variability. New advances in robotics and automated microscopy techniques have also increased the feasibility of high-throughput screenings based on *in situ* hybridization or immunostaining methods [149]. Moving a step further from the analysis of gene expression patterns, technological developments now allow large-scale monitoring of more complex phenotypic features; for example, MALDI-imaging mass spectrometry can produce metabolomics data with high spatial resolution [150–153]. Even the behaviour of whole organisms can be analysed using automated video tracking of confined [154] or free-living [155] animals. Together, these high-throughput approaches permit assessing phenotypes in a standardized and replicable manner.

Though it is often assumed that causality runs from genotype to phenotype, phenomics characterization could reveal causal links between *phenotypic* layers (e.g. increased risk of lung cancer or type II diabetes due to tobacco addiction or obesity, respectively), thereby overriding the need for genetics to act as the preferential level of causal explanation [3]. New synthetic approaches are starting to allow the direct manipulation of specific phenotypic levels while exerting a minimal perturbation on the global biological system, turning the search for causality beyond genetics into a more practicable endeavour. Optogenetics, the experimental control of protein function mediated by light, enables modulating cellular pathways with high spatio-temporal resolution in a quantitative manner [156]. Thus, it is now possible to trigger cell polarization [157], alter neuronal physiology in real time [158] or induce tissue morphogenesis [159] with precisely targeted techniques in intact organisms. These features include the possibility to persist in an out-of-equilibrium state, the engagement in cycles of growth and division, and, with particular relevance to the present discussion, the potential to evolve. Thus, these synthetic cellular systems could be used to explore cell-level mechanisms that might be acting as evolutionary constraints.

A comprehensive understanding of genotype-to-phenotype mapping will also necessitate a mechanistic description of how regulatory networks integrate the enormous range of environmental stimuli that shape phenotypic outputs. The fact that environmental inputs can modify developmental programmes and lead to altered phenotypes, a process called phenotypic plasticity, has been deeply studied by ecologists and geneticists [160,161]. Developmental biology, however, has historically paid less attention to this concept [162], focusing instead on the reverse phenomenon of canalization: how developmental programmes buffer environmental (and genetic) variation to reproducibly generate phenotypes. Hence, several mechanisms have been identified that allow developmental programmes to reject environmental perturbations [163], while explanations for how these programmes might instead *leverage* environmental cues are still scarce [164].

An underlying assumption of experimental biology is that the laboratory environment allows researchers to control external conditions and thus standardize the effect of the environment on the system under study. This approach, however, does not minimize the environmental dependency of the analysed phenomenon; it just provides a set of standard conditions that are used to compare different experiments from which more general rules and principles are then derived. Thus, many conclusions obtained from laboratory experiments do not hold when they are tested in natural environments (reviewed in [165]). If the biological processes under scrutiny are the mechanisms through which genotypes lead to phenotypes, which, as mentioned above, are known to be strongly dependent on environmental factors [160,161], restricting the external conditions to the laboratory environment seriously impairs our understanding of the phenomenon.

For these reasons, we advocate a deeper exploration of external conditions when studying genotype-to-phenotype mapping, beyond the optimal laboratory environment and closer to the actual stimuli that the studied organisms can find in their natural environments. Focusing on systems in their natural context with functional biology is gaining traction in the evolutionary community [166]. We propose that pairing such approaches with systematic manipulations imported from synthetic biology would provide new insights into evolutionary developmental biology. For example, unbiased characterization of genomic response to external signals would involve testing libraries of synthetic circuits under an extensive variety of tightly controlled environmental conditions that emulate realistic settings for the host organism. Far from being limited to controlled laboratory experiments, these studies could be carried out on multi-scale platforms such as Ecotrons [167,168].

Environmental effects on phenotype could also be systematically and selectively interrogated through synthetic interventions that trigger, disable or modify signalling pathways naturally linked to selective stimuli, such as nutrient-sensitive pathways [169], oxygen-sensing mechanisms [170] or light-controlled circadian signalling [171]. This approach is not often coupled with extensive phenotypic quantification, as the target pathway is typically identified for its ability to modulate a particular behaviour of interest, rather than for ‘pleiotropic’, knock-on, or incremental effects across the whole organism. By combining such methods with phenomics, a deeper understanding of how specific external factors propagate across phenotypic levels could be obtained.

Phenomics approaches will produce huge datasets that vary significantly in content as well as the method and framework of acquisition. How to integrate information and extract meaningful conclusions from diverse, high-dimensional datasets may become one of the most urgent biological challenges in the near future (reviewed in [172,173]). Addressing this challenge will require (i) developing standard methods for acquiring and processing different types of data, which will enable future automation; (ii) stimulating data sharing and data reuse in order to avoid duplication of efforts; and (iii) developing next-generation and community-oriented data platforms to facilitate data accessibility and standardization of processing methods [174]. Only by overcoming these limitations will it be possible to draw biological conclusions from phenomic measurements.

Collectively, the technologies and methods discussed here would allow researchers to empirically test the principles of genotype-to-phenotype mapping through a wide variety of synthetic approaches coupled with phenomic characterizations and controlled exposure to a range of realistic selective stimuli. The proposed experimental pipeline could identify and modulate contributing factors across biological scales and phenotypic levels up through environmental interactions, thereby providing a framework for the causal understanding of adaptive responses. Furthermore, the ability to measure multiple phenotypic levels in individual organisms will permit correlating stochasticity or variability across organizational scales, illuminating an otherwise elusive means by which phenotypic variation may be generated [175–177]. Systematic applications of the suggested techniques could ultimately help us distinguish between contingent limitations of known regulatory mechanisms and real biophysical constraints, which will be essential to provide evolutionary biology with predictive power.

## 5. Uniting synthetic and evolutionary biology: metabolism as a case study

In the previous sections, we have outlined synthetic approaches to perturb individual regulatory regions and GRNs, as well as emerging technologies for manipulating and characterizing phenotypes at the level of cells, tissues or organisms. Here, we demonstrate the joint potential of these approaches using metabolism as a case study. Metabolic networks—which have been the subject of intense study by both evolutionary and synthetic biologists—are more complex than GRNs, as they are not only regulated by transcription factors, but also respond to changes in the concentrations of metabolites that are themselves part of the network. This additional regulatory layer is essential for quick responses to a constantly changing extracellular environment [178,179]. Thus, metabolic processes are particularly suitable for examining genotype-to-phenotype mapping, as they couple regulatory networks that are hardwired in the genome (i.e. the levels and spatial distribution of enzymes or metabolic regulators) with external factors that are essential for the survival of the organism, such as nutrient availability. Though complex, the topology and dynamics of metabolic pathways are well characterized at the molecular and mathematical levels [180], permitting computational modelling and experimental manipulation to a higher degree than most biological systems.

Explaining the mechanisms that could originate such intricate networks constitutes a major question for evolutionary biologists. Although metabolic engineering has been mostly driven by industrial motivations (specifically the high cost of chemical synthesis), the quest to optimize bacteria or yeast strains to produce commercially viable compounds has also provided insight into the biophysical constraints, and thus the evolutionary potential, of metabolic networks. Metabolic engineering ranges from single genetic alterations that enhance an endogenous metabolic route [181,182] to the construction of entirely synthetic circuits that lead to compounds not produced by the wild-type strain [183]. However, the complex intertwinement of metabolic networks often leads to unexpected or undesired outcomes, limiting the scope of genetic engineering efforts to manipulate metabolism [184]. Instead, yield optimization is achieved by the iterative selection of the desired phenotypes. The success of selective breeding is subject to evolutionary constraints affecting the yield of biosynthetic processes, including the level of genomic plasticity that a particular prokaryote ‘species’ can tolerate [185,186] and the robustness of metabolic flux [187–189]. Hence, metabolic engineering combined with selective breeding (directed evolution) appears to be a well-suited approach to study evolutionary mechanisms, as it provides an experimental framework to explore how altered genotypes affect fitness-related phenotypes, to identify bottlenecks and possible ways to overcome them, and to quantitatively address system-level features such as complexity and optimality.

A comprehensive understanding of metabolism may be even more relevant to studying evolution in multi-cellular than unicellular organisms, given that metabolic processes, in addition to managing the bioenergetic requirements of cells, can directly alter signalling pathways and developmental programmes (reviewed in [190,191]). For example, the intracellular concentration of specific metabolites has been shown to regulate key ontogenetic processes such as zygotic genome activation [192] or the development of the presomitic mesoderm in vertebrates [193,194]. Thus, environmentally modulated metabolic states could impact a wide range of fitness-related phenotypes through pathways at all levels from embryonic development to organismal behaviour.

In sum, as technological advances increase our throughput to measure metabolism [3,195], we anticipate that synthetic perturbations to metabolic pathways could be used to explore functional evolution across animal populations under realistic environmental conditions. The combined use of synthetic approaches and phenomics offers an unprecedented opportunity to investigate how metabolic networks integrate genetics and external inputs into coherent phenotypic outputs, shedding light on the basic principles and mechanisms that underlie adaptive responses to environmental challenges.

## 6. Synthesis at scale: prospectus in synthetic evolutionary biology

Deep comprehension of the mechanisms behind genotype-to-phenotype mapping is essential for predicting adaptive evolution. Charting the map will require a concerted effort to blend mathematical theory and computational modelling with large-scale experiments yielding high-dimensional quantitative data. Synthetic approaches are a particularly promising avenue for empirically testing ideas inferred from genomic and morphological studies of extinct and extant lineages. The ability to manipulate individual genetic sequences has already produced insights into the origins of pleiotropy at the regulatory and network levels. Advancements in high-throughput methods for synthetic perturbation and phenomic measurement promise to elucidate macroscale morphological and behavioural changes relevant to natural selection. The sizable resource investment required by this ‘synthetic synthesis’ will be justified to produce new, qualitatively diverse data sufficient to identify not only causal factors behind specific phenotypes but also general rules for how perturbations to genotype are propagated across phenotypes. In this way, we might finally begin to develop a detailed understanding of the multi-scale interactions and constraints that shape the evolvability of biological systems.

Despite recent successes mapping targeted genomic perturbations to phenotypic outcomes, we have yet to harness the full potential of experimental evolution combined with synthetic approaches. For practical reasons, most existing efforts have focused on microbial systems, including a study of almost 600 *E. coli* strains that found network linkages synthetically introduced into endogenous GRNs tend to enhance adaptability to selection pressures [196]. Yet many more ongoing studies in microbial evolution could still benefit from synthetic augmentation. For example, only two of 58 possible cross-feeding interactions in *E. coli* have been observed to evolve under experimental conditions, despite the fact that the metabolic rewiring required to generate these two interactions is theoretically no less complex than to generate the other outcomes [197]. The ability to synthetically rewire elements of the metabolic network for one or both strains offers a controlled way to compare the performance of various cross-feeding pairs and systematically characterize environmental constraints, as well as to investigate the extent to which evolutionary innovations in one lineage trigger speciation in cohabiting lineages.

Synthetic approaches appropriate for microbial studies provide direct inroads into controlled experiments involving multi-cellular organisms. Gut microbiota are one avenue of particular interest, as they are the subject of significant medical attention as well as a growing body of evolutionary work [198,199]. One study in *D. melanogaster* proposed that microbiota contribute directly to speciation in host organisms [200], but subsequent small-scale experiments have failed to consistently replicate the results or indicate a clear mechanistic connection [201]. The combination of libraries of bacteria harbouring synthetic metabolisms with simulated ecological conditions could clarify the real extent and ecological relevance of this phenomenon.

As technological and theoretical progress continues, it will be crucial to extend the application of synthetic approaches to experimental evolution in multi-cellular organisms. A straightforward first step involves using mutational screens in transgenes or endogenous loci to generate sexually reproducing populations with varying degrees of genetic variation, in order to systematically quantitate how heterozygosity and genetic diversity influence evolutionary fitness. Given several starting populations homozygous at the allele(s) of interest, it should be possible to quantify the effects of heterozygosity in all possible pairwise couplings before moving to mixed populations of three or more lineages combined. This sequential, ‘tiered’ approach could be especially important for teasing out combinatorial effects, such as the recent observation that larval phenotypes in annelids result from the interplay of three genomes: maternal, paternal and zygotic [142]. Mixed populations of alleles could then be experimentally evolved under various selection pressures to link the degree of genetic diversity in a population to the dynamics of evolutionary adaptation.

Simultaneously, synthetic approaches should be leveraged to deepen our understanding of how GRNs evolve, which will be crucial for interrogating emergent features of complex multi-cellular organisms. For example, the flexible relationship between network structure and function affords greater potential for phenotypic variation than has been described in natural systems. While individual modules of fixed topology may be constrained in their evolvability, it appears that larger or more complex networks may be less constrained, due to the presence of redundant or ‘buffering’ mechanisms as well as the combinatorial increase in possible compensatory mechanisms. Future experiments with fully synthetic networks will permit more detailed research into the evolutionary potential of GRNs that interact to varying degrees with endogenous components at different times during the life cycle of an organism. Key to such studies is the ability to construct topologies unencumbered by evolutionary lineage and to sample a larger range of parameter space than could be expected to persist in a naturally evolving population. Eventually, the general principles derived from such experiments could be used to build circuits that directly influence fitness, in order to systematically investigate the evolvability of biological networks under controlled selection regimes.

Finally, multi-cellular organisms present unique challenges and opportunities for evolutionary study that should be both considered and capitalized upon. Notably, animals and plants possess a plethora of cell types and tissues that differ in behaviour almost exclusively due to differences in gene regulation [202]. Phenomic approaches in experimental evolution will be particularly useful to quantify changes in multiple tissues across many individuals in a population and therefore to trace the degree to which cells that share genetic material evolve independently. Here, too, synthetic reconstitution of cell-type-specific pathways might help to decouple intrinsic constraints on network function from constraints that arise internally due to shared usage of protein and regulatory apparatuses across different cell types within the same organism. In the future, more sophisticated synthetic manipulations to developmental programmes could shape the morphology of species and hence their environmental interactions, in order to explore macroscale selection pressures including physical or (eu)social considerations that may be irrelevant to microscopic species. Improvements to Ecotrons or other simplified ecosystems will further allow the methods proposed in this article to extend to community-level studies of several multi-cellular species evolving in tandem.

Though their motivations differ, evolutionary and synthetic biologists are both concerned with the mechanisms that decode genetic variation into phenotypic variation. Inspired by this convergence, we have illustrated here just a few of the ways in which synthetic approaches may shed light on outstanding problems in the evolution of genetic regulation by transcription factors. The framework we propose applies equally well to synthetic interventions modulating protein function [203–205], genome topology [206], cell-to-cell communication [207,208], post-transcriptional regulation [209,210] and neural behaviour [211,212], with increasing versatility as researchers continue to develop techniques for genetic engineering at scale. These studies will provide insight into basic evolutionary principles that will in turn enhance our ability to implement rational designs for living systems. Thus might we close the feedback loop between selected and synthesized, furnishing the variation of perspective from which human ingenuity has been, and is being, evolved.

## Data Availability

This article has no additional data.
